# Pure Tone Audiometry and Hearing Loss in Alzheimer's Disease: A Meta-Analysis

**DOI:** 10.3389/fpsyg.2021.788045

**Published:** 2022-01-21

**Authors:** Susanna S. Kwok, Xuan-Mai T. Nguyen, Diana D. Wu, Raksha A. Mudar, Daniel A. Llano

**Affiliations:** ^1^Carle Illinois College of Medicine, University of Illinois Urbana-Champaign, Urbana, IL, United States; ^2^Department of Speech and Hearing Sciences, University of Illinois Urbana-Champaign, Urbana, IL, United States; ^3^Department of Molecular and Integrative Physiology, University of Illinois Urbana-Champaign, Urbana, IL, United States; ^4^Beckman Institute for Advanced Science and Technology, University of Illinois Urbana-Champaign, Urbana, IL, United States; ^5^Carle Neuroscience Institute, Carle Foundation Hospital, Urbana, IL, United States

**Keywords:** pure-tone audiogram, Alzheimer's disease, age-related hearing loss, dementia, peripheral hearing, pure tone audiometry

## Abstract

An association between age-related hearing loss (ARHL) and Alzheimer's Disease (AD) has been widely reported. However, the nature of this relationship remains poorly understood. Quantification of hearing loss as it relates to AD is imperative for the creation of reliable, hearing-related biomarkers for earlier diagnosis and development of ARHL treatments that may slow the progression of AD. Previous studies that have measured the association between peripheral hearing function and AD have yielded mixed results. Most of these studies have been small and underpowered to reveal an association. Therefore, in the current report, we sought to estimate the degree to which AD patients have impaired hearing by performing a meta-analysis to increase statistical power. We reviewed 248 published studies that quantified peripheral hearing function using pure-tone audiometry for subjects with AD. Six studies, with a combined total of 171 subjects with AD compared to 222 age-matched controls, met inclusion criteria. We found a statistically significant increase in hearing threshold as measured by pure tone audiometry for subjects with AD compared to controls. For a three-frequency pure tone average calculated for air conduction thresholds at 500–1,000–2,000 Hz (0.5–2 kHz PTA), an increase of 2.3 decibel hearing level (dB HL) was found in subjects with AD compared to controls (*p* = 0.001). Likewise, for a four-frequency pure tone average calculated at 500–1,000–2,000–4,000 (0.5–4 kHz PTA), an increase of 4.5 dB HL was measured (*p* = 0.002), and this increase was significantly greater than that seen for 0.5–2 kHz PTA. There was no difference in the average age of the control and AD subjects. These data confirm the presence of poorer hearing ability in AD subjects, provided a quantitative estimate of the magnitude of hearing loss, and suggest that the magnitude of the effect is greater at higher sound frequencies.

**Systematic Review Registration:** https://www.crd.york.ac.uk/prospero/, identifier: CRD42021288280.

## Introduction

As the sixth leading cause of death in the United States, Alzheimer's disease (AD) affects nearly 6.2 million Americans (Alzheimer's Association., 2021). Projections of the aging population show a steep increase in this number to roughly 13.8 million by the year 2060 (Alzheimer's Association., 2021). The hallmark of AD is loss of episodic memory (McKhann et al., 2011). Over time, both increasing aggregation and spread of hyper-phosphorylated tau and β-amyloid protein throughout the brain result in memory, visuospatial, executive, personality, and language deficits (Small, 2000; McKhann et al., 2011; Reed et al., 2014). Progressive debilitation caused by this neurodegeneration carries substantial burden related to direct costs (i.e., hospitalizations, skilled nursing care, home health care, and hospice) and indirect costs (i.e., caregiver burden and diminished quality of life) (Burns, 2000; Reed et al., 2014; Arijita et al., 2017). To date, no cure exists for AD (McKhann et al., 2011). However, early treatments such as cholinesterase inhibitors and memantine may be used to slow the progression of AD symptoms (Anand and Singh, 2013; Sharma, 2019). Furthermore, lifestyle modifications such as increased aerobic activity, treatment of comorbid conditions as well as modifiable risk factors such as ARHL may slow progression of AD and lessen its impact on individuals and caregivers as secondary and tertiary prevention strategies (Khalsa, 2015; Hubbard et al., 2018; Jongsiriyanyong and Limpawattana, 2018; Mattson and Arumugam, 2018; Bhatti et al., 2020; Gregory et al., 2020).

The link between age-related hearing loss (ARHL) and the subsequent development AD is increasingly well-documented; however, the nature of this relationship remains unclear (Gurgel et al., 2014; Loughrey et al., 2018; Panza et al., 2018; Ray et al., 2018; Chern and Golub, 2019; Jafari et al., 2019; Ralli et al., 2019; Mertens et al., 2020; Utoomprurkporn et al., 2020; Knopke et al., 2021). Current hypotheses postulate that hearing loss increases cognitive demand and therefore predisposes individuals to AD neurodegeneration; that hearing loss results in social isolation which is a risk factor for AD; or that ARHL is an early clinical feature of AD pathology (Loughrey et al., 2018; Chern and Golub, 2019; Jafari et al., 2019; Ralli et al., 2019; Mertens et al., 2020; Utoomprurkporn et al., 2020; Knopke et al., 2021). Regardless of etiology, diagnosis of ARHL in AD may be useful since its treatment shows potential for being a modifiable risk factor to delay disease onset or slow rate of cognitive impairment (Hubbard et al., 2018; Jafari et al., 2019; Gregory et al., 2020; Mertens et al., 2020; Utoomprurkporn et al., 2020). As AD remains incurable, promotion of healthy lifestyle and reduction of modifiable risk factors remain the most practical and cost-effective methods of addressing the disease (Khalsa, 2015; Hubbard et al., 2018; Jongsiriyanyong and Limpawattana, 2018; Mattson and Arumugam, 2018; Bhatti et al., 2020; Gregory et al., 2020). To reduce the negative impacts of ARHL on AD, determination of whether treatment of peripheral auditory processing, central auditory processing, or some combination of both, is necessary (Ralli et al., 2019; Xu et al., 2019; Jayakody et al., 2020; Johnson et al., 2021; Knopke et al., 2021). Characterization of peripheral and central hearing ability in patients with AD using a variety of assessment modalities is crucial for understanding the relationship between ARHL and AD (Xu et al., 2019; Jayakody et al., 2020). Especially as the relationship between peripheral and central auditory processing in relation to AD remains unclear (Swords et al., 2018). Additionally, this information is necessary to determine the validity of quantitative measures of hearing ability and whether these metrics are correlated to other characteristics of AD (Hubbard et al., 2018).

Despite the many epidemiological studies that suggest a link between ARHL and the later development of AD (Uhlmann et al., 1989; Strouse and Hall, 1995; Quaranta et al., 2014; Bidelman et al., 2017; Haggstrom et al., 2018; Jayakody et al., 2018, 2020; Panza et al., 2018; Ray et al., 2018; Brewster et al., 2020; Sardone et al., 2021), fewer cohort studies have found a statistically significant difference in pure tone hearing thresholds between AD and control subjects (Wang et al., 2005, 2007; Gimeno-Vilar and Cervera-Paz, 2010; Idrizbegovic et al., 2011; Lin et al., 2011, 2013; Lodeiro-Fernandez et al., 2015; Villeneuve et al., 2017; Haggstrom et al., 2018; Hardy et al., 2019). Most of these studies had small sample sizes and were underpowered when detecting a difference in hearing thresholds (Wang et al., 2005, 2007; Gimeno-Vilar and Cervera-Paz, 2010; Idrizbegovic et al., 2011; Lin et al., 2011, 2013; Lodeiro-Fernandez et al., 2015; Villeneuve et al., 2017; Haggstrom et al., 2018; Hardy et al., 2019). On the contrary, greater impairments in audiological measurements related to central auditory processing, typically assessed using dichotic hearing tasks or electrophysiologic techniques such as electroencephalography (EEG), are increasingly reported (Grimes et al., 1987; Verma et al., 1987; Uhlmann et al., 1989; Schwartz et al., 1996; Revonsuo et al., 1998; Reeves et al., 1999; Pekkonen et al., 2001; Ally et al., 2006; Muscoso et al., 2006; Gates et al., 2008; Kimiskidis and Papaliagkas, 2012; Hsiao et al., 2014; Kurt et al., 2014; Iliadou et al., 2017; Shahmiri et al., 2017; Cintra et al., 2018; Morrison et al., 2018; Swords et al., 2018; Danjou et al., 2019; Mansour et al., 2019; Jafari et al., 2020; Tarawneh et al., 2021; Wang et al., 2021). Compared to central auditory processing, peripheral hearing is less expensive and less invasive to test and treat; therefore examining pooled data from studies that measure peripheral hearing ability in AD may provide useful insights (Wang et al., 2005, 2007; Gimeno-Vilar and Cervera-Paz, 2010; Idrizbegovic et al., 2011; Lin et al., 2011, 2013; Lodeiro-Fernandez et al., 2015; Villeneuve et al., 2017; Haggstrom et al., 2018; Hardy et al., 2019). Pure-tone audiometry is a “gold standard” procedure that is universally used to objectively measure and classify hearing ability (Wang et al., 2005, 2007; Gimeno-Vilar and Cervera-Paz, 2010; Idrizbegovic et al., 2011; Lin et al., 2011, 2013; Lodeiro-Fernandez et al., 2015; Villeneuve et al., 2017; Haggstrom et al., 2018; Hardy et al., 2019). PTA uses pure tone stimuli in the range of 250 to 8000 Hz to assess air conduction hearing thresholds and measures the lowest intensity at which tones are perceived at least 50% of the time (Wang et al., 2005, 2007; Gimeno-Vilar and Cervera-Paz, 2010; Idrizbegovic et al., 2011; Lin et al., 2011, 2013; Lodeiro-Fernandez et al., 2015; Shahmiri et al., 2017; Villeneuve et al., 2017; Haggstrom et al., 2018; Hardy et al., 2019; Mansour et al., 2019). Unlike other audiologic assessments, PTA has been shown to be effective at measuring hearing ability even in those who are cognitively impaired (Liberati et al., 2009). The current meta-analysis seeks to use pooled data collected from published studies identified through PRISMA guidelines for systematic review, to characterize the peripheral hearing ability of subjects with AD measured by pure tone audiometry (Liberati et al., 2009). Quantification of the degree of hearing loss in AD subjects relative to normal hearing controls will help to understand the burden of hearing loss in these patients and to plan interventions that target ARHL.

## Methods

### Search Strategy

A systematic review of literature using PRISMA guidelines was conducted on studies published prior to August 18th, 2021 using PubMed, Cochrane Library, Web of Science, and Scopus databases (Liberati et al., 2009). The search strategy was created with consultation with an expert medical and biomedicine librarian at the University Library at the University of Illinois—Urbana, Champaign. The search was implemented by one searcher (SSK). A non-MeSH search term: “pure tone” AND “(Alzheimer^*^ OR dementia)” resulted in a total of 417 studies; no languages were excluded. All citations containing information on author, title, source, and full abstract were exported from each database as a BibTeX file and uploaded to Mendeley® citation manager for preliminary review. Once duplicates were removed, the abstracts, methods, and results of 248 studies were individually screened. Off-topic studies were removed first and the remaining studies were assessed using the inclusion/exclusion criteria set by this study. This was completed by one rater (SSK) and repeated by two raters (XMTN and DDW) for validation.

To be included for analysis, articles had to contain full-text featuring a cohort-study design that involved subjects diagnosed with AD compared against a control group, both with exclusion criteria of comorbid hearing disorders or deafness. These studies had to report pure-tone air conduction measurement pure tone average or threshold (dB) at a specified frequency (Hz). Studies that did not meet these criteria were rejected on initial review. Studies that categorized subjects based on hearing ability or studied the use of hearing aids or implants were excluded from this study using the PRISMA 2020 flow protocol for new systematic reviews of searches conducted in only databases and registers (Page et al., 2005), and this modified PRISMA flow diagram was generated using ReviewManager©5.4, a software developed by Cochrane© Reviews.

**Figure 1:**
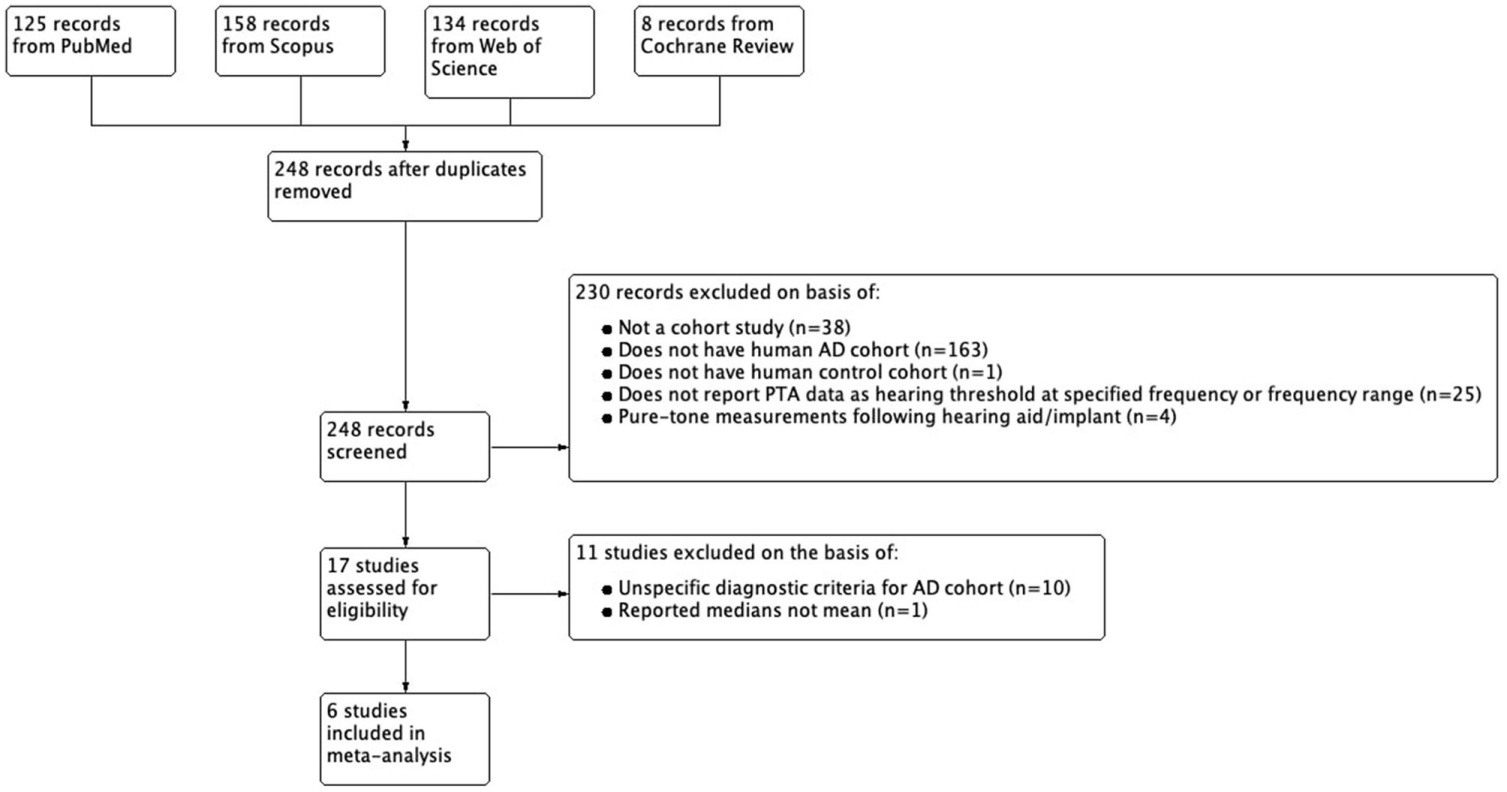
Flowchart of inclusion and exclusion of studies.

Of the 17 studies that met initial inclusion and exclusion criteria, 11 were excluded on the basis of having unclear diagnostic methods or criteria to define their AD cohort; 1 of these was excluded due to statistically significant differences in age between their AD cohort and control; and 1 study was excluded for reporting pure-tone measures in medians and not means. Diagnostic criteria for the AD cohort had to involve physician assessment of subject medical history, physical/neurological examination, and neuropsychological assessment. Studies with more specific criteria for AD cohort such as the use of NINCDS-ADRDA criteria, neuroimaging, and cerebrospinal fluid analysis were also included. 6 studies met criteria for our final analysis including 4 written in English, 1 written in Spanish, and 1 written in simplified Chinese. The non-English studies were translated into English using translation software from Google®. Figure 1 summarizes the selection process. The methods listed above were conducted by one searcher/rater (SSK) with consultation with a medical and biomedicine librarian. These methods were repeated by two raters (XMTN and DDW) for validation.

### Data Extraction

Following the identification of included studies summarized in Figure 1, full-text PDF articles were downloaded for independent, in-depth screening by three authors (SSK, RAM, and DAL). The following information was extracted from each study if available: author, year published, criteria defining subjects with AD, total number of subjects with AD, sex ratio of subjects with AD, mean age with standard deviation, pure-tone audiogram frequency and/or range of frequencies and associated hearing threshold linked to ear tested, and neurocognitive testing scores. Additional metrics were extracted (such as race, ethnicity, highest level of education attained, etc.), but due to limited reporting they could not be further analyzed. Due to variation related to the ear used for pure-tone audiometry (studies reported from left, right, combined, or better ear), mean thresholds from the “better ear” were used. These data were also collected for the control cohort of each study. Data reported as a threshold frequency (Hz) for a specific pure-tone audiogram frequency or pure-tone audiogram frequency range was extracted directly onto an Excel® spreadsheet. For studies that reported this information using a 2D graph, WebPlotDigitizer© 4.4 was used to extract thresholds (Hz) from the y-axis to their corresponding frequency from the x-axis. All graphs were uniquely calibrated using to their pre-existing scale without correction. Pure tone audiometry thresholds were averaged for 500–1,000–2,000 Hz (referred to here as 0.5–2 kHz PTA) and separately for 500–1,000–2,000–4,000 (referred to here as 0.5–4 kHz PTA).

### Risk of Bias

Studies were assessed for potential bias using the Newcastle-Ottawa criteria for cohort studies by one rater (SSK) (Reeves et al., 2011; Wells et al., 2013). Each study was assessed independently in three categories: Selection, Comparability, and Outcome. Selection criteria assessed the representativeness of the AD cohort, the control cohort, certainty of diagnosis, and demonstration that hearing loss was not used to select for or against inclusion into the study. A maximum of four stars can be given in this domain. The comparability domain assesses whether a study controlled for confounding variables such as age, sex, and other factors. This domain can receive a maximum of one star. Lastly, the outcome domain, with a maximum score of three stars, measures studies based on whether independent-blinding was used and whether follow-up duration was sufficient to measure outcomes and complete follow-up for all subjects was assessed. Table 1 summarizes the Newcastle-Ottawa scores for each study that was included in the meta-analysis. These results were verified with discussion between two raters (RAM and DAL).

**Table 1:** Risk of bias scores calculated for included studies using the Newcastle Ottawa Scale for Cohort Studies.

**References**	**Selection**	**Comparability**	**Outcome**
**Newcastle-Ottawa assessment of bias for cohort studies**			
Gates et al. (1995)	⋆⋆⋆	⋆	⋆⋆
Gimeno-Vilar and Cervera-Paz (2010)	⋆⋆⋆⋆	⋆	⋆
Hardy et al. (2019)	⋆⋆⋆	⋆	⋆
Idrizbegovic et al. (2011)	⋆⋆⋆⋆	⋆	⋆
Wang et al. (2005)	⋆⋆⋆⋆	⋆	⋆
Wang et al. (2007)	⋆⋆⋆	⋆	⋆

### Statistical Analysis

Meta-analysis was conducted in Cochrane© Review's software, Review Manager 5.4, and recapitulated in R© to generate figures used in this paper. A random-effects model was applied for meta-analysis due to the heterogeneity of methods in each study. This model was selected over a fixed-effects model because our study design pooled different independent studies from a heterogeneous population. Therefore, to allow for the true effect to vary across subjects due to differences such as age, gender, race, highest and level of education achieved, we defined our combined subject populations as a random sample with a relevant distribution of effects. The combined effect estimates from our meta-analysis estimates the mean effect in this distribution instead of assuming that all of the individual study populations from each study had a single homogenous true effect size. Aggregate outcome data across the six studies were continuous in nature and only pooled study means were used for the data analysis, not individual-level data. We utilized pooled study means rather than individual level means because the latter was not publicly available in the published studies that were included in this study. The summary statistic used in this meta-analysis was the mean difference. It was assumed that the variation in standard deviation (SD) between studies reflected differences in the reliability of the outcome measurements and not differences in outcome variability in the study populations. By doing this, studies with a small SD are given relatively higher weight while studies with larger SD are given relatively smaller weights. The weight given to each of the 6 studies was determined by the inverse-variance method to assign a quantitative value to how much influence each study has on the overall results of the meta-analysis. Inverse-variance is determined by taking the inverse of the variance of the effect estimate for each study (i.e., one over the square of its standard error). Therefore, more weight is given to studies with more precision compared to those with lower precision. Subgroup analysis was conducted for frequency ranges of 500–1,000–2,000 Hz (0.5–2 kHz PTA) and 500–1,000–2,000–4,000 Hz (0.5–4 kHz PTA). Differences in means between the AD and control groups were conducted using a two-tailed *t*-test with *p* < 0.05 considered statistically significant. A t-test was used in this study to examine if hearing threshold was different among those with AD compared to controls. This analysis was completed for 0.5–2 kHz PTA and 0.5–4 kHz PTA. The mean hearing threshold difference between 0.5–2 kHz PTA and 0.5–4 kHz PTA was determined using an unpaired, two-tail t-test.

## Results

A total of six studies, totaling 171 (102 females) subjects with AD and 222 (135 females) control subjects (healthy aging or subjective memory complaints), were included in data analysis. Not all studies reported neurocognitive testing outcomes. For subjects with AD, only four studies reported mean Mini-Mental State Examination (MMSE) data, and these scores were combined as the summary MMSE score. For the control cohort, only three studies reported mean MMSE data which were averaged as the collective MMSE score. Average MMSE score for AD subjects = 19 ± 4.3 (SD), and average MMSE score for control subjects = 27 ± 2.1 was significantly higher (*p* < 0.001). The demographic characteristics for each study included in the meta-analysis is summarized in Table 2.

**Table 2:** Demographics from each included study for subjects with Alzheimer's disease and control subjects.

**References**	**Alzheimer's disease cohort**			**Control (Normal/SMC) cohort**		
	**Number of subjects (female)**	**Mean age ± SD (years)**	**Mean MMSE ± SD**	**Number of subjects (female)**	**Mean age ± SD (years)**	**Mean MMSE ± SD**
Gates et al. (1995)	20 (10)	78.3 ± 6.5	N/A	40 (23)	76.5 ± 7.5	N/A
Gimeno-Vilar and Cervera-Paz (2010)	14 (9)	79.0 ± 6.0	N/A	14 (9)	76.0 ± 5.0	N/A
Hardy et al. (2019)	20 (9)	69.4 ± 8.1	18.6 ± 5.9	34 (15)	66.7 ± 6.3	N/A
Idrizbegovic et al. (2011)	43 (23)	64.3 ± 6.4	24.5 ± 4.8	34 (22)	64.0 ± 5.1	29.0 ± 1.0
Wang et al. (2005)	31 (10)	73.1 ± 7.5	15.0 ± 3.6	50 (33)	73.3 ± 6.6	26.2 ± 2.7
Wang et al. (2007)	43 (13)	72.7 ± 6.4	17.9 ± 3.1	50 (33)	73.3 ± 6.6	26.3 ± 2.5

Due to the known positive correlation between age and hearing-loss (Loughrey et al., 2018; Panza et al., 2018; Ray et al., 2018; Chern and Golub, 2019; Jafari et al., 2019; Ralli et al., 2019; Mertens et al., 2020; Utoomprurkporn et al., 2020; Knopke et al., 2021), a pooled, standardized mean age difference was calculated. Using a random-effects model, a DerSimonian-Laird meta-analysis for mean age difference across all studies was conducted. This analysis, which weighted studies based on standard-deviation, showed that the aggregated AD cohort had a mean increase in 0.70 years compared to their respective control cohort. This difference was not statistically significant (*p* = 0.292).

**Figure 2:**
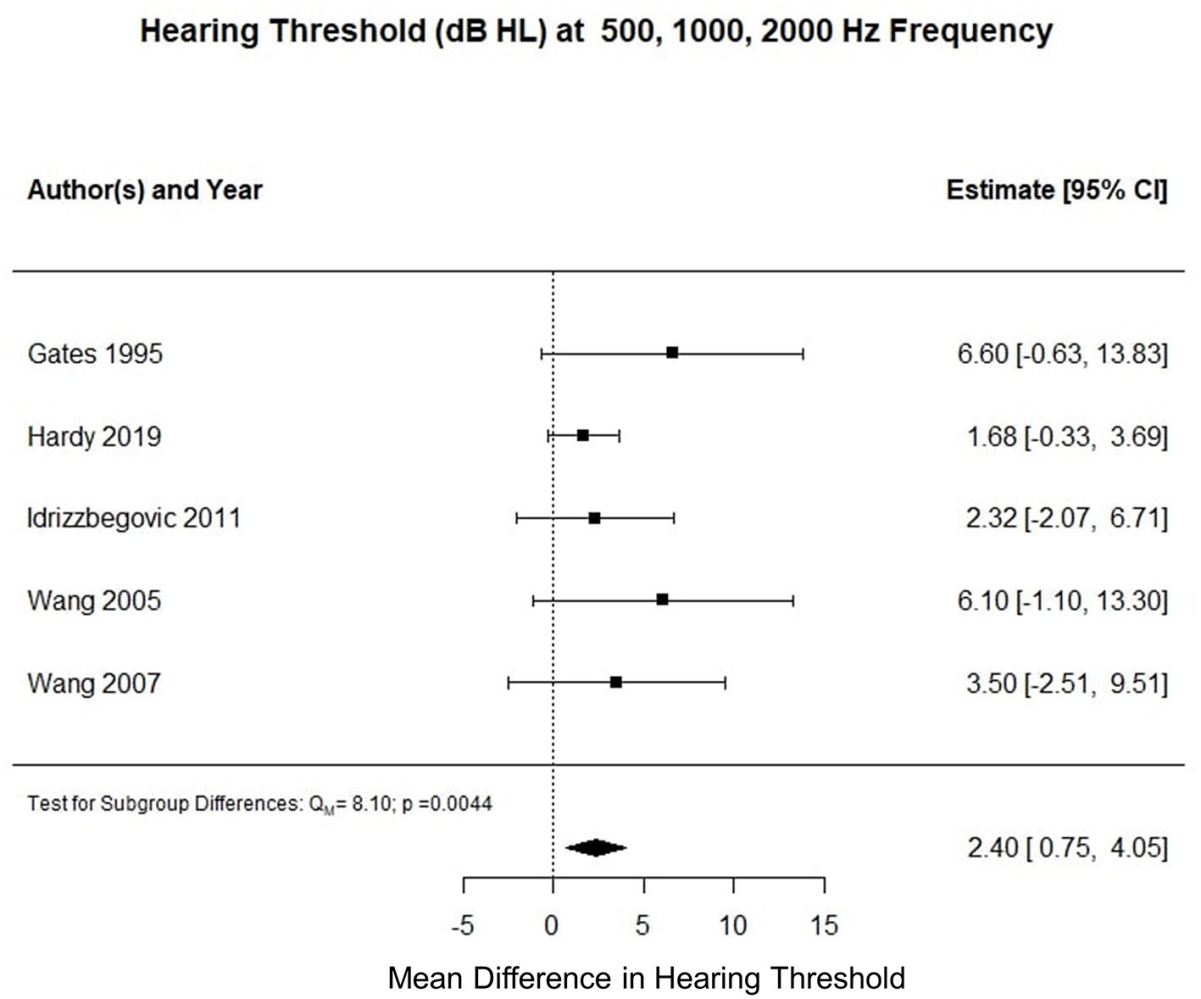
Forest plot summarizing meta-analysis findings of hearing threshold (dB HL) difference between subjects with Alzheimer's disease and control subjects at 0.5–2 kHz PTA.

Five studies reported three frequency pure tone audiometry calculated for air conduction thresholds at 500, 1,000, and 2,000 Hz (0.5–2 kHz PTA). The meta-analysis of their means showed that the AD cohort had a 2.3 decibel hearing level (dB HL) higher compared to the control cohort (*p* < 0.001). Figure 2 summarizes the findings of the meta-analysis conducted for hearing threshold (dB HL) averaged at 0.5–2 kHz PTA.

**Figure 3:**
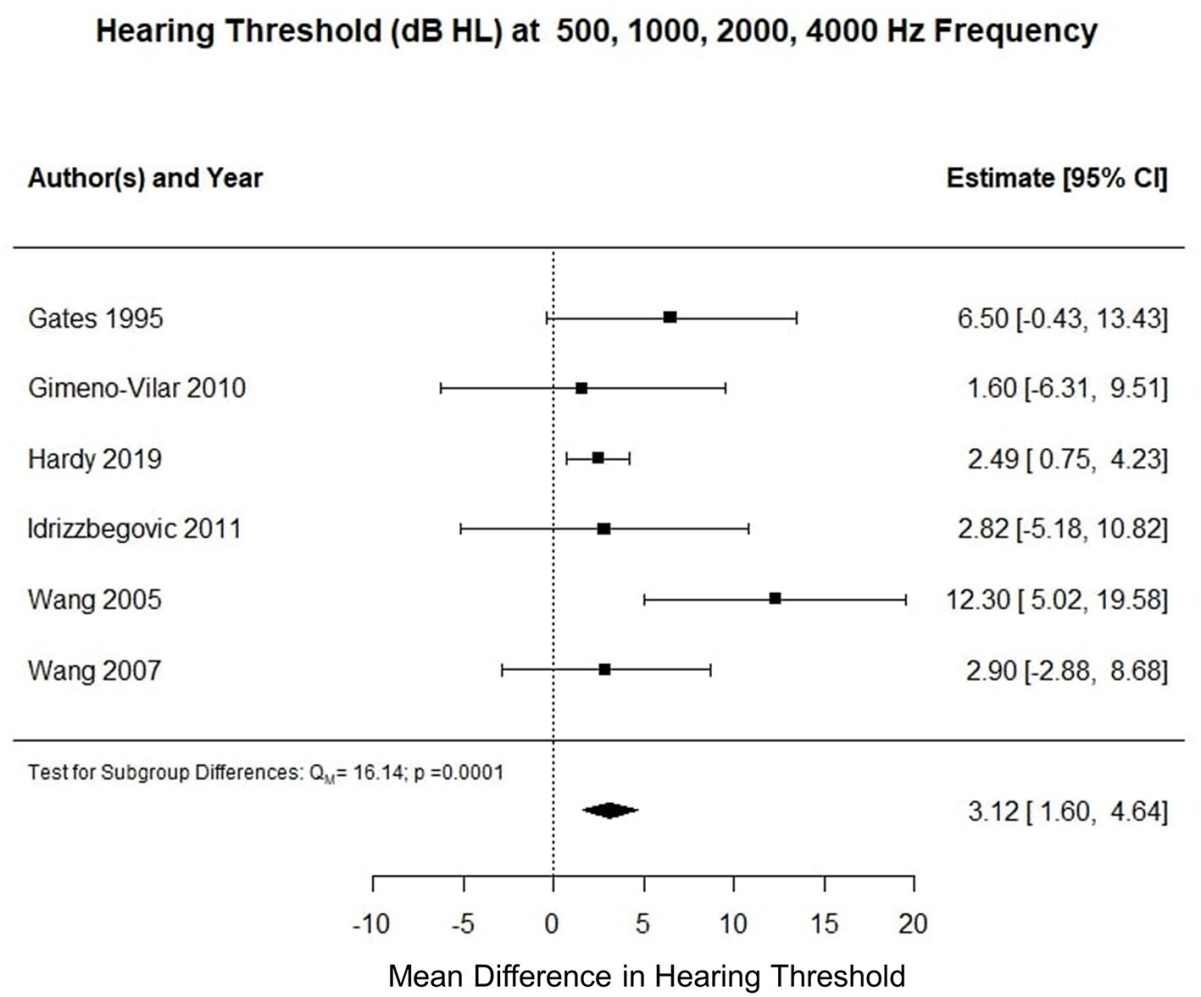
Forest plot summarizing meta-analysis findings of hearing threshold (dB HL) difference between subjects with Alzheimer's disease and control subjects at 0.5–4 kHz PTA.

Six studies reported pure tone audiometry calculated for air conduction thresholds at 500, 1,000, 2,000, and 4,000 Hz (0.5–4 kHz PTA). The meta-analysis of their means showed that the AD cohort had a 4.5 decibel hearing level (dB HL) higher compared to the control cohort (*p* < 0.002). Figure 3 summarizes the findings of the meta-analysis conducted for hearing threshold (dB HL) at 0.5–24 Hz PTA. Using an unpaired t-test, the mean difference between hearing threshold for 0.5–2 kHz PTA and 0.5–4 kHz PTA was −2.66 dB (t = 22.849, df = 326, standard error of difference = 0.116, *p* < 0.001).

## Discussion

We observed a statistically significant increase in hearing thresholds, measured by pure tone audiometry at frequency ranges from 0.5 to 2 kHz (2.3 dB difference) and 0.5–4 kHz (4.5 dB difference), in subjects with AD compared to similar-aged controls. We found the difference in hearing thresholds at these two frequency ranges to be statistically significant where hearing threshold measured at 0.5–4 kHz PTA was greater by 2.66 dB than thresholds measured using 0.5–2 kHz PTA. These data suggest that AD is associated with an up to 4.5 dB hearing loss in frequencies associated with normal speech communication and that the loss of hearing is increased at higher frequencies.

### Limitations of the Current Study

The methodology of this study was limited having a sole searcher (SSK) to identify potential studies to be included in the meta-analysis. Although the search methods were repeated by two other independent raters for verification (XMTN and DDW), having an initial search conducted by one individual may have introduced selection bias to this study. This meta-analysis was further limited by the small number of studies that met eligibility criteria. Several studies were excluded as they did not characterize AD specifically. In addition, few studies quantified peripheral hearing ability using pure tone audiometry thresholds at specific frequencies. Third, included studies were heterogeneous in their methods and reported outcomes that limited analysis to a small number of shared outcomes. Important subject characteristics such as highest level of education received, pre-retirement occupation, race, ethnicity, socio-economic status, medications taken, number and type of co-morbid conditions, number of years from initial diagnosis, and many other factors that could account for differences between groups was not reported in the majority of studies and therefore could not be analyzed in our meta-analysis. Moreover, history of noise exposure was not reported in any of the 6 studies used for this analysis. However, all 6 studies excluded subjects on the basis of hearing disorder diagnosis and deafness. Additionally, included studies varied in their definition of AD and their control population (i.e., some studies used a healthy-aging cohort while others used subjects with subjective memory complaints). However, despite these limitations which would be expected to dilute the impact of AD on hearing loss, we observed a statistically-significant increase in hearing threshold in AD subjects. Due to the aforementioned heterogeneity of reported results and lack of reported results in each individual study included in this analysis, it is possible that variables other than age and dementia impacted the difference in hearing ability of subjects with AD vs. controls. Although we sought to identify the potential for publication bias using the Newcastle Ottawa scale, we acknowledge that each study included in this meta-analysis featured an individual positive correlation between hearing loss and AD, which combined provided a pooled positive correlation. We cannot ignore the propensity of studies to report only positive findings, and therefore, we acknowledge that our meta-analysis may not have captured studies with negative findings on the relationship between hearing loss and AD, as no such studies were found in our search.

There were 11 studies that were excluded from this meta-analysis due to the criteria established in the Methods section (Gates et al., 2008; Lodeiro-Fernandez et al., 2015; Bidelman et al., 2017; Villeneuve et al., 2017; Jayakody et al., 2018; Gyanwali et al., 2020; Sardone et al., 2020, 2021; Utoomprurkporn et al., 2020; Aylward et al., 2021; Jung et al., 2021). Of these, all but one demonstrated findings of subjects with dementia having increased hearing thresholds (i.e., hearing impairment) compared to controls; however, the findings of Haggstrom et al. (2018) could not be used in our analysis as they reported threshold hearing loss as median-values and not mean-values. Overall, the trend of increased hearing loss in subjects with dementia compared to controls was present in both included and excluded studies, supporting both the findings of this meta-analysis and current literature (Loughrey et al., 2018; Panza et al., 2018; Ray et al., 2018; Chern and Golub, 2019; Jafari et al., 2019; Ralli et al., 2019; Mertens et al., 2020; Utoomprurkporn et al., 2020; Knopke et al., 2021).

The shortcomings outlined above illustrate the importance of better characterizing peripheral hearing ability in subjects with AD compared to healthy-aging controls. Further work should characterize peripheral hearing ability using pure-tone audiometry in individuals diagnosed with clearly defined criteria for AD relative to cognitively normal age-, sex-, and education-matched controls. The findings of peripheral hearing assessment in such studies should be compared to measures of central auditory processing by carefully considering factors such as years since diagnosis, co-morbid factors, and cognitive factors such as global cognitive function, verbal language comprehension. This approach will serve to not only improve our understanding of the correlation between ARHL and AD, but also determine targets for early intervention to slow the progression of AD.

### Implications of This Study in Relation to Previous Work

A large number of epidemiological studies have reported a relationship between ARHL and the later development of cognitive impairment. In a systematic review of 17 articles, Thomson et al. found pure-tone audiometry to be the most commonly reported method of quantifying ARHL in patients with dementia; in each of the studies they analyzed, all demonstrated an association between hearing loss and increased incidence of dementia (Thomson et al., 2017). Similarly, a meta-analysis conducted by Taljaard et al. (2015) showed reduced cognitive function in subjects with untreated hearing loss with the degree of cognitive function positively correlated to the degree of hearing impairment (Taljaard et al., 2015). Several prospective studies have also demonstrated risk of incident dementia increasing with worsening hearing loss measured by pure tone audiometry (Wang et al., 2005, 2007; Gimeno-Vilar and Cervera-Paz, 2010; Idrizbegovic et al., 2011; Lin et al., 2011, 2013; Lodeiro-Fernandez et al., 2015; Bidelman et al., 2017; Villeneuve et al., 2017; Haggstrom et al., 2018; Hardy et al., 2019; Chern et al., 2021). Although the underlying mechanism for this relationship remains unclear, reduction of peripheral hearing ability has been correlated to increased beta-amyloid deposition (Ray et al., 2018; Chern et al., 2021) and cortical thinning of the left frontal, right temporal, and bilateral occipital regions of the brain which suggests an important relationship between peripheral hearing and central processing in relationship to neuropathology (Uhlmann et al., 1989; Iliadou et al., 2017; Ha et al., 2020).

Conversely, some studies have not observed a correlation between ARHL and AD. For example, a meta-analysis conducted on 36 studies found that ARHL quantified by pure-tone audiometry was associated with cognitive impairment in general, but this association was not found between peripheral hearing loss and specifically for AD (Loughrey et al., 2018). The authors mention that smaller small size may have contributed to this insignificant association. Additionally, the relationship between peripheral ARHL and AD is complicated by the interactions of the peripheral and central auditory systems (Panza et al., 2018). Although the field of audiology often refers to PTA as a method of quantifying peripheral hearing ability, it is undeniable that auditory sensation, processing, and perception requires conductive, sensorineural, and central processing which is described in depth by authors of this study in their previous work (Swords et al., 2018). However, following the convention of previous audiologic studies of AD subjects, we refer to PTA as a metric of predominantly peripheral hearing. Therefore, to elucidate the relationship between peripheral and central auditory perception in relation to AD, PTA should be used to assess hearing ability alongside methods that quantify central auditory processing such as auditory evoked potentials.

In the current study a relatively small increase in threshold (2.3–4.5 dB HL) was observed in the AD cohort relative to control. Although the clinical significance of this magnitude of difference is not yet known, it is important to note that this degree of hearing loss roughly corresponds to the thresholds for detecting amplitude modulations in sounds (Scheider and Pichora-Fuller, 2001). Loss of amplitude modulation detection may lead to deficits in speech perception in AD (Page et al., 2005), which may contribute to the well-documented deficits in central auditory processing in AD (Swords et al., 2018).

## Conclusion

To our knowledge, this is the first meta-analysis providing quantification of peripheral hearing loss measured by pure tone audiometry for subjects with AD compared to age-matched controls. Our findings suggest that subjects with AD have higher hearing thresholds at 0.5–2 kHz PTA and 0.5–4 kHz PTA compared to age-matched controls. This finding is supported by current literature from epidemiological studies on the relation of ARHL and AD. Our meta-analysis suggests that in future studies peripheral hearing should be better characterized in AD cohorts compared to age-matched controls accurately estimate the contributions of peripheral hearing loss to cognitive impairment. In addition, to use peripheral hearing loss in AD as a modifiable risk factor, assessing hearing ability using pure-tone audiometry on a routine basis would be critical. Additionally, analyses comparing pure tone audiometry measurements to other characteristics associated with AD may yield improved understanding on the effects of peripheral hearing in AD and elucidate effects of confounding variables that could not be analyzed in this study. Lastly, to characterize the pathophysiologic relationship between age-related hearing loss and AD, future studies should utilize pure-tone audiometry alongside other audiologic and neurophysiologic measures of peripheral and central hearing.

## Data Availability Statement

Publicly available datasets were analyzed in this study. All data are publically available in the publications cited in the article.

## Author Contributions

SK performed the systematic literature review, determined study inclusion/exclusion, extracted and analyzed data, and prepared the manuscript. X-MN validated study methods, performed data analysis, prepared the manuscript, and edited the manuscript. DW validated study methods, prepared figures, and edited the manuscript. RM determined study inclusion/exclusion criteria, validated extracted data, and prepared and edited the manuscript. DL determined study inclusion/exclusion, validated study methods, validated extracted data, and prepared and edited the manuscript. All authors contributed to the article and approved the submitted version.

## Funding

RM was supported by an ASH Foundation New Century Scholars Research Grant. DL was supported by an award from the Benjamin R. and Elinor W. Bullock and Edwin E. and Jeanne Bullock Goldberg Professorial Scholar Fund.

## Conflict of Interest

DL has consulted for Eisai Pharmaceuticals in the past year. The remaining authors declare that the research was conducted in the absence of any commercial or financial relationships that could be construed as a potential conflict of interest.

## Publisher's Note

All claims expressed in this article are solely those of the authors and do not necessarily represent those of their affiliated organizations, or those of the publisher, the editors and the reviewers. Any product that may be evaluated in this article, or claim that may be made by its manufacturer, is not guaranteed or endorsed by the publisher.
